# Analysis of Dot/Icm Type IVB Secretion System Subassemblies by Cryoelectron Tomography Reveals Conformational Changes Induced by DotB Binding

**DOI:** 10.1128/mBio.03328-19

**Published:** 2020-02-18

**Authors:** Donghyun Park, David Chetrit, Bo Hu, Craig R. Roy, Jun Liu

**Affiliations:** a Department of Microbial Pathogenesis, Yale University School of Medicine, New Haven, Connecticut, USA; b Microbial Sciences Institute, Yale University, West Haven, Connecticut, USA; c Department of Microbiology and Molecular Genetics, McGovern Medical School, The University of Texas Health Science Center at Houston, Houston, Texas, USA; Institut Pasteur

**Keywords:** secretion system, whole-cell tomography, nanomachine, protein transport, Dot/Icm system, effector proteins, *Legionella pneumophila*, type IV secretion, cryoelectron tomography

## Abstract

Type IV secretion systems (T4SSs) are sophisticated nanomachines used by many bacterial pathogens to translocate protein and DNA substrates across a host cell membrane. Although T4SSs have important roles in promoting bacterial infections, little is known about the biogenesis of the apparatus and the mechanism of substrate transfer. Here, high-throughput cryoelectron tomography (cryo-ET) was used to visualize Legionella pneumophila T4SSs (also known as Dot/Icm secretion machines) in both the whole-cell context and at the cell pole. These data revealed the distribution patterns of individual Dot/Icm machines in the bacterial cell and identified five distinct subassembled intermediates. High-resolution *in situ* structures of the Dot/Icm machine derived from subtomogram averaging revealed that docking of the cytoplasmic DotB (VirB11-related) ATPase complex onto the DotO (VirB4-related) ATPase complex promotes a conformational change in the secretion system that results in the opening of a channel in the bacterial inner membrane. A model is presented for how the Dot/Icm apparatus is assembled and for how this machine may initiate the transport of cytoplasmic substrates across the inner membrane.

## Abstract (precis)

Many bacteria use type IV secretion systems (T4SSs) to translocate proteins and nucleic acids into target cells, which promotes DNA transfer and host infection. The Dot/Icm T4SS in Legionella pneumophila is a multiprotein nanomachine that is known to translocate over 300 different protein effectors into eukaryotic host cells. Here, advanced cryoelectron tomography and subtomogram analysis were used to visualize the Dot/Icm machine assembly and distribution in a single L. pneumophila cell. Extensive classification and averaging revealed five distinct intermediates of the Dot/Icm machine at high resolution. Comparative analysis of the Dot/Icm machine and subassemblies derived from wild-type cells and several mutants provided a structural basis for understanding mechanisms that underlie the assembly and activation of the Dot/Icm machine.

## INTRODUCTION

Type IV secretion systems (T4SSs) are used by many bacteria to translocate proteins and nucleic acids into target cells, which promotes DNA transfer and host infection. T4SSs are multiprotein nanomachines that span the cell membranes of Gram-negative and Gram-positive bacteria (1–4). Phylogenetically, T4SSs can be classified into two subtypes, known as the IVA and IVB secretion systems. The IVA secretion systems are represented by the Agrobacterium tumefaciens VirB/D system and Escherichia coli R388 and pKM101 plasmids (5, 6). The Dot/Icm systems present in Legionella pneumophila, Coxiella burnetii, and Rickettsiella grylli are prototypical IVB secretion systems (7). Because the T4SS machines are major virulence determinants that are involved in human diseases and antibiotic resistance, they have been identified as promising therapeutic targets (8).

Structural details for several IVA machines have furthered our understanding of how these systems function. Xanthomonas citri VirB/D-, Helicobacter pylori Cag-, and E. coli R388-encoded IVA secretion machine structures resolved by single-particle cryoelectron microscopy (cryo-EM) and X-ray crystallography revealed that the IVA secretion machines are composed of an outer membrane-associated core complex (OMCC) that includes a large periplasmic assembly of proteins that connects to the secretion channel in the outer membrane (9–13). The OMCC recruits integral inner membrane (IM) proteins that connect to a cytoplasmic complex. Single-particle cryo-EM analysis of the E. coli R388 IVA secretion machine showed that the cytoplasmic complex is arranged in a double-barrel structure composed of two hexamers of the VirB4 ATPase, and the VirD4 ATPase has been found to localize between these two barrels (11, 14).

The IVB secretion machines have been characterized by visualizing intact nanomachines using cryoelectron tomography (cryo-ET). Visualization of the Dot/Icm machine in L. pneumophila revealed the OMCC as a triangular WIFI structure (see Fig. S1A in the supplemental material) (15–17). This structure presumably includes the OM-associated DotC, DotD, and DotH proteins and IM-associated proteins DotG and DotF. Similar structural features were observed for the IVB OMCC and the IVA OMCC (15–18). Unlike the E. coli R388-encoded IVA secretion machine, however, the cytoplasmic complex of the L. pneumophila Dot/Icm machine forms a central secretion channel made of an IM-anchored DotO ATPase (VirB4-related) complex that consists of a hexamer of DotO dimers. The DotB (VirB11-related) ATPase is dynamic, and, presumably, one important stage that is necessary for translocation of substrates by the Dot/Icm machine is the recruitment of a DotB hexamer from the cytosol to the type IVB complex through a direct interaction with the DotO complex (15). Recently, *in situ* cryo-ET structures of the Cag secretion system in H. pylori and the F plasmid conjugation system in E. coli (19, 20) provided evidence that the VirB4-related ATPases in these systems assemble a structure that is similar to that of the DotO complex.

10.1128/mBio.03328-19.1 FIG S1 Characterization of the L. pneumophila Dot/Icm type IVB secretion system. (A) A cartoon model depicting the L. pneumophila Dot/Icm type IVB secretion system (T4SS). T4SSs are mostly found on cell poles. The periplasmic complex is composed of several structural units: wheel, plug, disk, collar, and cylinder. The cytoplasmic complex is composed of DotO and DotB ATPases as described by Chetrit et al. (15). The location of IM-associated coupling protein DotL remains unknown. (B) Average dimensions of L. pneumophila (*N* = 42) in the stationary phase. Magenta defines poles, and yellow defines lateral region. Values are means ± standard errors of the means (SEM). (C) Quantification of the polar and lateral L. pneumophila T4SSs. Error bars represent SEM. Data were compared using a *t* test. Download FIG S1, PDF file, 0.1 MB. Copyright © 2020 Park et al. 2020 Park et al. This content is distributed under the terms of the Creative Commons Attribution 4.0 International license.

Currently, there is little known about how these machines are constructed in the bacterial cell and how conformational changes may accompany or promote substrate transfer by the type IV apparatus. To address these questions, structures for L. pneumophila Dot/Icm machines were obtained *in situ* by utilizing a high-throughput cryo-ET pipeline (21) for a multiscale imaging analysis.

## RESULTS

### Whole-cell visualization of Legionella pneumophila Dot/Icm machines.

**FIG 1:**
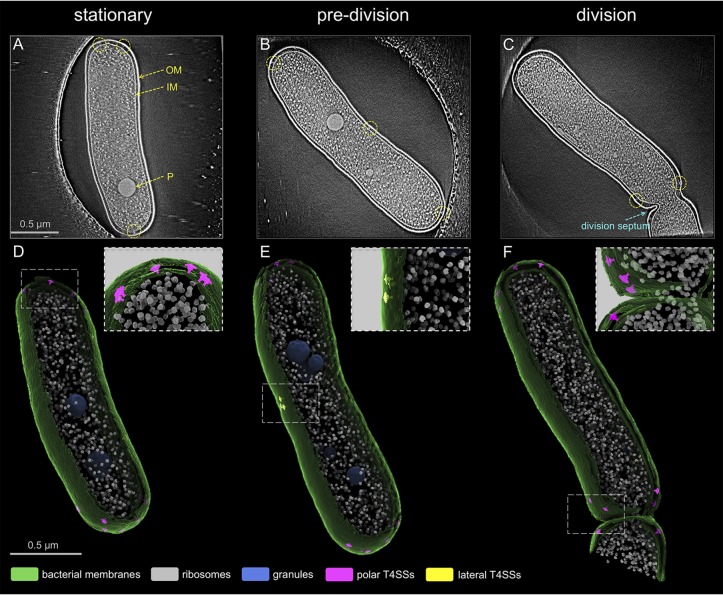
Localization pattern of individual L. pneumophila T4SS machines. (A to C) Central sections of tomograms showing entire L. pneumophila cells at different stages of bacterial growth: stationary, elongation, and division phase. Panels are arranged in a progressing order of bacterial growth. Outer membrane (OM), inner membrane (IM), and granule (P) are annotated. Dotted circles indicate the T4SS machines. (D to F) 3D renderings of the tomograms shown in panels A to C. The upper right corner insets represent magnified views of boxed regions.

The L. pneumophila Dot/Icm machines are located primarily at the poles of the bacteria, and polar localization of the Dot/Icm machine is thought to be important for intracellular replication (18). Previous cryo-ET studies have focused on imaging cell poles to enrich the number of Dot/Icm machines for *in situ* structural analysis (15–17); however, the distribution pattern of individual Dot/Icm machines and their assembly in the context of a whole cell remains to be defined in detail. To address this question, low-magnification (pixel size, 5.4 Å/pixel) cryo-ET tilt series were collected, and tomograms of the intact wild-type (WT) cells at different stages of bacterial growth were generated (Fig. 1; see also Movie S1 in the supplemental material). With advanced imaging tools, such as the Volta phase plate (VPP) (22) and direct electron detector with energy filter, the Dot/Icm machines were visualized in the context of a whole cell (Fig. 1A to C). Bacterial cells in stationary phase showed uniform dimensions of ∼1.8 ± 0.03 μm long and ∼0.5 ± 0.004 μm wide (Fig. 1A and D and Fig. S1B). Bacteria growing exponentially in log phase included elongated cells that were in a predivision phase (Fig. 1B and E) and cells that just entered the division phase that had a clear division septum (Fig. 1C and F).

10.1128/mBio.03328-19.9 MOVIE S1 3D rendering of a tomogram showing an entire L. pneumophila cell. Download Movie S1, MOV file, 5.8 MB. Copyright © 2020 Park et al. 2020 Park et al. This content is distributed under the terms of the Creative Commons Attribution 4.0 International license.

Previously, fluorescence microscopy analysis demonstrated that some Dot/Icm machines are targeted to the lateral mid-cell region at an early stage of cell division, and this division site then becomes the new pole after septation (18). Data here show a trend similar to that of Dot/Icm machine localization; however, a small number of lateral Dot/Icm machines (∼1.6 per cell) were also found to be present in stationary-phase cells (Fig. S1C). Subsequently, the number of lateral Dot/Icm machines increases to an average of ∼3.0 during the predivision phase (Fig. S1C). Detailed examination of 82 poles from stationary-phase cells revealed that ∼8.1 Dot/Icm machines, on average, were present on each pole of a WT L. pneumophila cell (Fig. S1C), while the previous estimation was ∼4.2 Dot/Icm machines on each pole of L. pneumophila cells (18). Importantly, a considerable amount of structural heterogeneity of the Dot/Icm machines was evident within individual cells (Fig. S2A), which suggested that these structures represent different subassemblies of the Dot/Icm machine. Subtomogram averaging and classification of 1,164 Dot/Icm machines from the low-magnification whole-cell tomograms resulted in two distinct class averages: (i) incomplete Dot/Icm structures (∼73%) that consisted of machines having OM-associated components but lacking associated cytoplasmic assemblies, and (ii) intact Dot/Icm structures (∼27%) that had OM, IM, and cytoplasmic components (Fig. S2B). Mapping back of these two class average structures into original tomograms showed that the two classes are found both in the pole and the mid-cell region within a L. pneumophila cell (Fig. S2C). Taken together, this whole-cell cryo-ET analysis suggested that there is an ordered process for assembling the Dot/Icm machine that occurs throughout the bacterial cell body.

10.1128/mBio.03328-19.2 FIG S2 Dynamic assembly of the L. pneumophila T4SS machine. (A) Snapshots of tomographic slices showing subassemblies of T4SS. (B) Two major subtomogram average classes found in whole-cell cryo-ET data. (C) Mapping back of the two class averages into original positions. Download FIG S2, PDF file, 0.2 MB. Copyright © 2020 Park et al. 2020 Park et al. This content is distributed under the terms of the Creative Commons Attribution 4.0 International license.

### Visualization of structural intermediates in the Dot/Icm assembly pathway.

To further define and obtain detailed structural information on potential subassemblies, we collected high-magnification (pixel size, 2.5 Å) cryo-ET tilt series and generated tomograms that focus on each cell pole. A total of 7,913 Dot/Icm subtomograms from L. pneumophila strains (WT, *dotB*_E191K_, Δ*dotB*, and Δ*dotL* strains) were aligned and analyzed by subtomogram averaging and classification (15). This analysis included structures obtained from a *dotB*_E191K_ strain, which is a strain producing a mutant version of the DotB protein that can bind ATP but has a defect in hydrolysis, and from the Δ*dotL* strain, which is a mutant deficient in the IM-associated coupling protein DotL. The *dotB*_E191K_ and Δ*dotL* strains were included in this analysis because the periplasmic and cytoplasmic complexes observed in these strains were similar to those found in the WT strain. Similarly, the Δ*dotB* mutant assembles a Dot/Icm machine that is similar to that detected in the WT strain, with the only previously noted difference being that the cytoplasmic density corresponding to the position of the DotB protein was absent from the structure.

**FIG 2:**
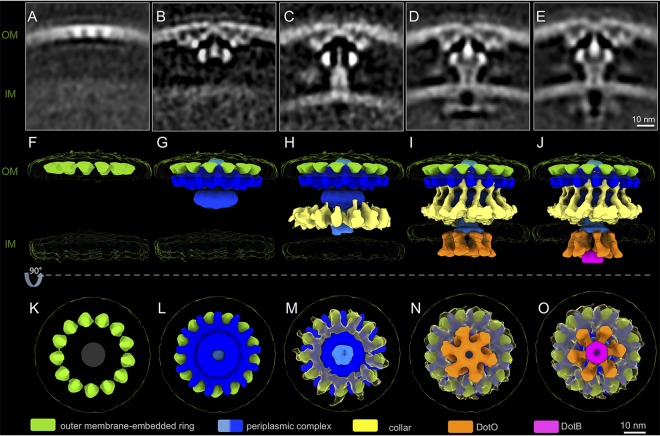
Unique structural intermediates suggest the “outside-inside” model of the L. pneumophila T4SS machine assembly. (A to E) Central sections of the class average structures showing distinct structural conformations. Panels are arranged in an order of increasing structural complexity. (F to O) 3D surface models of structural intermediates in panels A to E in side views (F to J) and bottom views (K to O).

These data revealed five distinct structures of the Dot/Icm machines, which were arranged according to increasing structural complexity (Fig. 2 and Fig. S3A and B). The smallest and least complex subassembly consisted of a ring structure displaying 13-fold symmetry embedded in the OM (Fig. 2A, F, and K). This structure has been named the outer membrane-embedded ring (OMER). The second subassembly has the wheel, plug, and periplasmic disk complex of the Dot/Icm machine attached to the OMER (Fig. 2B, G, and L and Fig. S1A). It has been suggested that the lipoprotein DotC associates with the OM and is critical for initiating the assembly of the Dot/Icm machine (17, 23). Thus, DotC is likely a principal component of the OMER, which serves as a nucleation site for the recruitment of the additional outer membrane-associated core complex (OMCC) proteins (12) (Fig. 2B, G, and L). The density corresponding to the IM appears smeared in this subassembly (Fig. 2B), suggesting that the OM-associated components have not engaged with the IM yet. In the third subassembly, there is a cylinder that surrounds the plug domain, and it appears to bridge the OMCC with a more clearly defined IM complex (Fig. 2C, H, and M). Comparing the second and third subassemblies, it is evident that there is a relocation of the plug domain that now lies below the disk attached to the OMCC to interact with the cylinder. As both plug and cylinder are intimately associated in the rest of the Dot/Icm machine, the plug-cylinder interaction may be critical for its assembly (Fig. S4A to C). Lastly, in this third structure, there is the recruitment of a cage-like structure that surrounds the machine, and a density corresponding to the IM is more evident than that of previous structures (Fig. 2C, H, and M). Thus, assembly of the primary elements of the core complex appears to be complete in this intermediate; however, there is no evidence that elements of the cytoplasmic complex have been recruited to the machine (Fig. 2C, H, and M).

10.1128/mBio.03328-19.3 FIG S3 Particle compositions of the five structural intermediates. (A) Central sections of the class averages showing district structural conformations. Panels are arranged in an order of increasing structural complexity. (B) Particle distribution charts corresponding to average structures shown in panel A. Download FIG S3, PDF file, 1.0 MB. Copyright © 2020 Park et al. 2020 Park et al. This content is distributed under the terms of the Creative Commons Attribution 4.0 International license.

10.1128/mBio.03328-19.4 FIG S4 Newly described structural features of the L. pneumophila T4SS machine. (A and D) A central section of an asymmetrically reconstructed average structure showing the L. pneumophila T4SS machine. (B and C) 3D representations of the boxed region in panel A highlighting the plug-cylinder association. (E) A cross-section along the *z* axis of the structure in panel D, highlighting the collar connection to the wheel. (F and G) Cross-section views at the positions indicated in panel D. (H) 3D representation of the periplasmic complex. (I to K) 3D representations of the collar. Download FIG S4, PDF file, 0.2 MB. Copyright © 2020 Park et al. 2020 Park et al. This content is distributed under the terms of the Creative Commons Attribution 4.0 International license.

Asymmetric reconstruction of the mature periplasmic complex revealed that the cage-like structure connects with the wheel and that both display 13-fold symmetry (Fig. S4D to K). The cytoplasmic ATPases DotO and DotB are recruited to the Dot/Icm machine in these last two subassemblies (Fig. 2D, E, I, J, N, and O). A hexamer of DotO dimers is visible in the fourth intermediate, and in the fifth intermediate, a hexamer of the DotB protein is docked beneath the DotO complex. Upon docking of the ATPase complexes, the distance between OM and IM changes from ∼35 nm to ∼30 nm in the two structures (Fig. 2C to E). The subassembly that does not have DotB associated with DotO (Fig. 2D, I, and N) has an architecture that is identical to that of the Dot/Icm machine visualized in a Δ*dotB* mutant of L. pneumophila (15). Because DotB is essential for the function of the Dot/Icm machine (24), this DotB-free subassembly represents a conformation of the Dot/Icm machine in an inactive state, whereas the last subassembly that has DotB bound to DotO likely represents a conformation that is in an early stage of Dot/Icm activation, in which the machine is not engaged with a recipient cell that will receive type IV substrates; therefore, the apparatus will not be translocating effectors.

We have previously shown by live imaging and fluorescence-based microscopy that DotB associates with the Dot/Icm machine at the cell pole through interactions that require DotO (15). The polarity of DotB is dependent on the stability of the OMCC protein DotC, the core complex protein DotG, and the inner membrane protein DotI. This result supports a biogenesis pathway in which assembly of an intact Dot/Icm machine is initiated by the OMER complex that nucleates formation of periplasmic core complex and inner membrane complex, which generates a machine capable of recruiting the cytoplasmic ATPases (15) (Fig. S5A and B). In addition, the polar targeting of the Dot/Icm machine was recently shown to be mediated by DotU and IcmF (17). The absence of these proteins, or the outer membrane subunit DotC or DotD, diminished DotG polarity. Consistent with these data, a deletion strain that did not produce the OMCC subunit DotC failed to recruit the periplasmic DotG-sfGFP subunit to the cell pole, whereas elimination of the inner membrane subunit DotI or the cytosolic subunit DotO or DotB did not affect polar localization of DotG-sfGFP (Fig. S5A and B). Furthermore, subtomogram averaging of isogenic deletion mutants lacking a specific subunit supports the hypothesis that an intact OMER is required for assembly of the periplasmic core complex, and that a periplasmic core complex is required for recruitment of the cytosolic ATPases DotO and DotB (Fig. S5C). Thus, these data support a hierarchal assembly pathway that is initiated by the OMER complex.

10.1128/mBio.03328-19.5 FIG S5 “Outside-inside” model of the L. pneumophila T4SS machine assembly. (A) Real-time visualization of DotB_E191K_-sfGFP or DotG-sfGFP in mutants deficient in the indicated Dot components. Bar, 3 μm. (B) Polarity scores of DotB_E191K_-sfGFP and DotG-sfGFP in the indicated deletion strains. The red horizontal lines represent the medians of the polarity scores (*n *= 200 cells for each strain; *, *P < *0.000, calculated by a two-tailed Mann-Whitney test). (C) Central sections of subtomogram averages of Dot-deficient mutants. Download FIG S5, PDF file, 0.2 MB. Copyright © 2020 Park et al. 2020 Park et al. This content is distributed under the terms of the Creative Commons Attribution 4.0 International license.

### Conformational changes in the cytoplasmic complex mediated by DotB binding.

**FIG 3:**
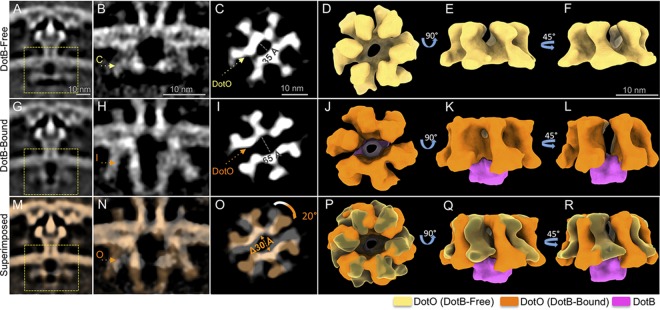
Docking of the DotB ATPase complex induces conformational change of the entire cytoplasmic complex. (A and G) Class average structures shown in Fig. 2D and E. (B and H) Central sections of asymmetrically reconstructed cytoplasmic complex structures of the DotB-free (B) and DotB-bound (H) intermediates. (C and I) Cross-section views at the positions indicated in panels B and H. (D to F) 3D renderings of the DotB-free cytoplasmic complex. (J to L) 3D renderings of the DotB-bound cytoplasmic complex. (M to R) Superimposition of the DotB-free and DotB-bound cytoplasmic complex.

The DotO-DotB energy complex is critical for substrate translocation by the Dot/Icm machine (8). There were apparent differences in the DotO complex when structures of the Dot/Icm machines that were associated with DotB were compared with structures where DotB was not associated with DotO (Fig. 2I and J). Focused refinement of the cytoplasmic complex of the DotB-free and DotB-bound subassemblies was conducted to determine the detailed conformational changes of the cytoplasmic complex induced by DotB. Without DotB bound, the cytoplasmic complex consists of a hexameric arrangement of DotO dimers that present six central domains forming an elliptical ring (Fig. 3A to F). This DotO complex displayed pseudo-2-fold symmetry along the major axis of the central ring, which is reminiscent of the pseudo-2-fold symmetry found in PilF and PilB ATPase complex structures of the type IV pilus system (25, 26). Aside from the unique geometry of the DotO complex, a high level of symmetry mismatch was observed throughout the secretion machine (Fig. S6). Strikingly, in structures where DotB was associated with the DotO complex, there was a 30-Å separation of the DotO complex along a pseudo-2-fold axis of symmetry (Fig. 3G to L). The three DotO dimers on each side of the area of separation remained intimately associated (Fig. 3J to L), and interactions with the DotB complex appeared to stabilize the two DotO subcomplexes to maintain the large assembly as a hexamer of DotO dimers (Fig. 3L). Additionally, binding of DotB to the DotO complex triggered an ∼20° clockwise rotation of the entire cytoplasmic complex (Fig. 3M to R).

10.1128/mBio.03328-19.6 FIG S6 Symmetry mismatch in the L. pneumophila Dot/Icm machine. Cross-section views at indicated positions of a 4× binned global average structure showing the entire apparatus (A to D) and a 2× binned focused refinement structure showing the cytoplasmic complex (E to I). Scale bars, 10 nm. Download FIG S6, PDF file, 0.1 MB. Copyright © 2020 Park et al. 2020 Park et al. This content is distributed under the terms of the Creative Commons Attribution 4.0 International license.

**FIG 4:**
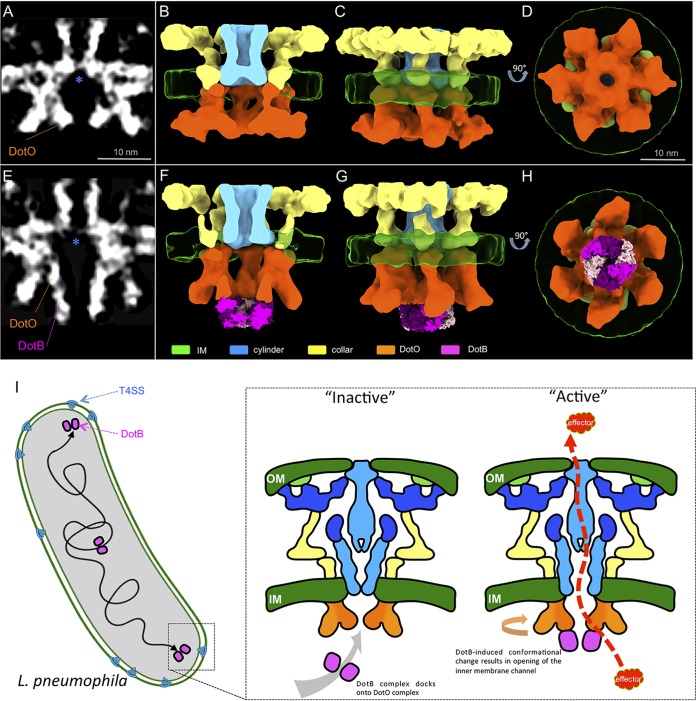
DotB-induced conformational change opens the type IV secretion channel. (A and E) Central sections of cytoplasmic complex structures of the DotB-free (A) and DotB-bound (E) intermediates. Blue asterisks highlight the location of the cytoplasmic end of the secretion channel. (B to D and F to H) 3D renderings of the averaged structure shown in panels A and D, respectively. Twofold symmetry was imposed along the axis of innate 2-fold pseudosymmetry to facilitate 3D segmentation. PDB entry 6GEB was docked in the place of DotB density. (I) Proposed model of L. pneumophila T4SS activation. Docking of DotB complex induces conformational changes that open the IM secretion channel for effector translocation.

To determine if additional conformational changes were induced upon DotB binding (Fig. 4A to H), the DotB-free and DotB-bound structures were further refined to ∼20.8-Å and ∼21.8-Å resolutions, respectively (Fig. S7). Importantly, the improved electron density map of the DotB complex bound to DotO was in close agreement with the X-ray crystal structure of the L. pneumophila DotB complex structure reported recently in regard to both the dimensions of the DotB complex and the appearance of a double-stacked ring density corresponding to the N- and C-terminal domains of DotB revealed in the crystal structure (Fig. 4E to H) (24). These data indicate that the DotB protein is directly engaged with the DotO complex to confer structural changes in the Dot/Icm apparatus and further validates the assignment of DotB and DotO to the structural densities obtained from these subtomogram averages.

10.1128/mBio.03328-19.7 FIG S7 Resolution estimation by ResMap. (A and B) Central sections of the DotB-free and DotB-bound cytoplasmic complex structures, respectively. (C to F) Local resolution estimation by ResMap. Download FIG S7, PDF file, 0.8 MB. Copyright © 2020 Park et al. 2020 Park et al. This content is distributed under the terms of the Creative Commons Attribution 4.0 International license.

### DotB binding to DotO opens an inner membrane channel in the Dot/Icm machine.

One of the most striking details revealed in these refined structures was the rearrangement of an apparent IM channel in the apparatus that is likely mediated by DotB binding to the DotO complex. The structure of the DotB-free subassembly included a V-shaped cylinder domain that lies above the DotO complex in the IM (Fig. 4A to D). In the absence of DotB bound to DotO, this channel appeared to be closed, with the tip of the V-shaped structure pointing toward the bacterial cytoplasm. A conformational change in this IM assembly was revealed when the DotB-free structure was compared to the DotB-bound structure. In the DotB-bound state, the closed V-shaped structure becomes an open cylinder and a transmembrane pore appeared in the IM (Fig. 4E to H). An association between the IM-embedded portion of the collar, cylinder, and DotO complex were observed in the DotB-free state (Fig. 4A to D), whereas docking of the DotB complex resulted in the dissociation of the DotO complex from these neighboring structural domains (Fig. 4E to H). These data demonstrate that the conformational changes to the cytoplasmic complex mediated by DotB binding to DotO are transduced to other components of the Dot/Icm machine, resulting in one of the most dramatic conformational changes: the opening of a channel in the IM (Fig. 4C, G, and I, and Movie S2). Importantly, these results are consistent with the hypothesis that DotB binding is essential for an early stage of effector translocation by the Dot/Icm machine (15).

10.1128/mBio.03328-19.10 MOVIE S2 Animation showing a plausible mechanism of L. pneumophila T4SS assembly and conformational changes induced by DotB binding. Download Movie S2, MOV file, 2.6 MB. Copyright © 2020 Park et al. 2020 Park et al. This content is distributed under the terms of the Creative Commons Attribution 4.0 International license.

## DISCUSSION

To understand the mechanism of type IVB secretion, the structure and dynamic recruitment of L. pneumophila Dot/Icm components was characterized recently using fluorescence microscopy and cryo-ET (15–17). Here, whole-cell cryo-ET and extensive classification were utilized to visualize distribution and intermediates of the Dot/Icm system. Specifically, VPP-enhanced whole-cell cryo-ET analysis significantly improved image contrast by boosting low-resolution frequency signals (22) and resulted in a better quantification and estimation of Dot/Icm distribution in a single bacterial cell. Additionally, extensive subtomogram classification of a large data set resulted in identification of five distinct intermediates of the Dot/Icm machine. These data have provided a structural basis to understand the mechanism of the Dot/Icm machine assembly and the role of the DotO-DotB-energizing center in promoting structural changes to the Dot/Icm machine.

It was shown previously that the lipoproteins DotC and DotD, together with DotH, are components of an OM-localized complex that is essential for assembly of the Dot/Icm machine (12, 15). Presumably the OMER structure, which is the smallest Dot/Icm subassembly identified, contains these essential determinants. Consistent with these proteins being components of the OMER, an OMER-like structure was identified when the proteins DotC, DotD, and DotH were expressed together with the proteins DotU and IcmF in a strain of L. pneumophila that had all the other *dot* and *icm* genes deleted (17). The proteins DotU and IcmF are IM determinants that localize to the cell pole to stabilize the Dot/Icm machine and enhance polar assembly. Thus, it is likely that polar localization of DotU and IcmF precedes recruitment of the OMER at the cell pole. Because the OMER structure was present in all five Dot/Icm subassemblies shown here, and given that OMER proteins are essential for machine assembly, these data indicate that the OMER is a platform that nucleates the assembly of this complex Dot/Icm machine. This supports a model where the Dot/Icm assembly process initiates at the OM. Based on the five states of Dot/Icm assembly, we propose a model where the OMER (stage 1) nucleates the assembly of periplasmic and transmembrane proteins that comprise the OMCC (stage 2). This facilitates organization of an IM complex that would include a secretion pore (stage 3), and the IM complex promotes assembly of the cytoplasmic energizing complex consisting of DotO (stage 4) and DotB (stage 5).

*In situ* structure of the Dot/Icm machine from a mutant of L. pneumophila that does not encode the DotO protein indicates that recruitment of the cytoplasmic ATPase complex is not required for the maturation of a periplasmic complex (15). Consistent with these data, analysis of the five structural intermediates visualized in L. pneumophila encoding a functional Dot/Icm machine demonstrated that the cytoplasmic ATPases were not recruited to the apparatus until the OMCC had been assembled. It is worth noting, however, that most of the particles that displayed an intact periplasmic complex also displayed the cytoplasmic complex, which suggests the ATPase assembly is rapidly recruited once the OMCC has been assembled (Fig. 2).

Previous data presented the cytoplasmic complex as a structure containing a DotO complex assembled as a hexamer of DotO dimers (15), which was in contrast to studies indicating that the DotO homolog in the Dot/Icm machines associate with the IM complex as two independent barrels (16). This unexpected structural feature of the cytoplasmic complex was obtained in part by applying 6-fold symmetry to the cytoplasmic complex to refine the structure, which raised the concern that this was not an accurate structural representation of the complex. Here, structures of the cytoplasmic complex were obtained in the state where DotB was not associated with DotO (DotB free) and in the state where DotB was associated with DotO (DotB bound). Sixfold symmetry was not applied to these structures, and this resulted in structures that showed features identical to those of the structure reported previously. Both structures identified a rosette-like arrangement of dimeric DotO subunits assembled in a hexameric conformation. The refined structure has pseudo-2-fold symmetry in the DotO complex, which was not apparent in the previous structure. It is likely that applying a 6-fold symmetry to the density map eliminated this unique feature. Importantly, pseudo-2-fold symmetry had been observed in hexamers of PilB and PilF, which are ATPases from the type IV pilus system (25, 26). Additionally, the structure of the DotB complex associated with the DotO complex matched with the crystal structure of the DotB complex determined recently by X-ray crystallography (24). Thus, it is unlikely that there are major anomalies introduced into the DotO-DotB structure that resulted from applying symmetry during the refinement stage of analysis.

The DotB protein is essential for the translocation of Dot/Icm substrates into host cells (8), and DotB binding to DotO likely represents an important stage in Dot/Icm activation. Consistent with this hypothesis, cryo-ET analysis of the intermediates of the Dot/Icm cytosolic complex revealed that the binding of DotB to DotO induced several conformational changes. The DotO complex itself was altered upon DotB binding. The central channel in the DotO complex measured 35 Å in diameter in the DotB-free state and was 65 Å in the DotB-bound state, which indicates that DotB binding widens the DotO central channel significantly. There was also a 20° clockwise rotation of the DotO complex observed upon DotB binding. Furthermore, one of the most dramatic changes observed in the Dot/Icm structure upon DotB binding was the opening of a channel that spans the IM, which is located above the central channel in the DotO complex. DotO is intimately attached to the IM complex, which would explain why conformational changes in DotO that are induced by DotB binding are transduced across the inner membrane and can change the overall conformation of the Dot/Icm machine. Importantly, this IM channel could provide a conduit that would allow translocated substrates of the Dot/Icm system to gain access to the central channel in the OMCC. The coupling protein complex containing the DotL protein has the potential to guide substrates into the IM channel via a long C-terminal extension on DotL that associates with effector-binding adapter proteins such as IcmS and IcmW (27, 28). Because there are systems, such as the R388-encoded Trw system, that do not encode a VirB11 (DotB) homolog, it is possible that substrate transfer can occur solely by a VirD4/VirB4-dependent mechanism (11). In this regard, the DotB-dependent opening of the IM channel may be an adaptation that enhanced the versatility of the type IV system to translocate substrates that have diverse biochemical properties. Future studies should reveal how the DotO-DotB complex functions to energize the complex to direct the translocation of effector proteins into host cells.

## MATERIALS AND METHODS

### Bacterial strains.

Bacterial strains used for this study are derived from L. pneumophila LP01 and LP02 strains (see Table S1 in the supplemental material), and each construct has been generated by standard recombinant DNA techniques as described previously (15, 29). L. pneumophila was grown on charcoal yeast extract (CYE) plates at 37°C (30).

10.1128/mBio.03328-19.8 TABLE S1 Strains used in this study. Download Table S1, PDF file, 0.1 MB. Copyright © 2020 Park et al. 2020 Park et al. This content is distributed under the terms of the Creative Commons Attribution 4.0 International license.

### Cryo-ET sample preparation.

Bacterial cultures were grown 48 h at 37°C on CYE agar plates. Bacteria were removed from the plate and suspended in water and then mixed with 10-nm colloidal gold particles for marker-dependent tilt series alignment. The mixture was deposited onto freshly glow-discharged holey carbon grids for 1 min, blotted with filter paper, and frozen in liquid ethane using a gravity-driven plunger apparatus as described previously (21, 31).

### Cryo-ET data collection and reconstruction.

The frozen-hydrated specimens were imaged with 300-kV electron microscopes. Cell pole data (high magnification) was collected using a Polara G2 electron microscope (FEI Company) equipped with a field emission gun and a direct detection device (Gatan K2 Summit). Tile series were acquired with a magnification of ×15,500, resulting in a pixel size of 2.5 Å at the specimen level as described previously (15). Whole-cell data (low magnification) was collected from single-axis tilt series at ∼1-μm defocus, with an accumulative dose of ∼60 e^−^/Å^2^ using a Titan Krios electron microscope (Thermo Fisher Scientific) equipped with a field emission gun, an energy filter, a Volta phase plate, and a direct detection device (Gatan K2 Summit). The tomographic package SerialEM (32) was utilized to collect 40 image stacks at a range of tilt angles, between −60° and +60° for each tilt series, at a magnification of ×26,000, resulting in a pixel size of 5.45 Å at the specimen level. Each stack contained 10 to 15 images, which were aligned using Motioncorr2 (33) and then were assembled into the drift-corrected stacks by TOMOAUTO (21). The drift-corrected stacks were aligned and reconstructed by IMOD marker-dependent alignment (34). In total, 68 tomograms (3,838 by 3,710 by 1,600) were generated. Binned tomograms (2 by 2 by 2) were used for 3D segmentation, and 4-by-4-by-4 binned tomograms were used to direct image analysis. SIRT reconstruction was used for segmentations and direct image analysis. WBP reconstruction was used for the subtomogram averaging.

### Classification of *Legionella* growth and cell dimension measurement.

From the tomograms acquired at ×26,000 magnification, we classified cells based on features such as their cell length and the presence of a division septum. We found a population of cells that showed highly consistent dimensions (Fig. S1B), which, based on cell length, indicated that the cells were in stationary phase (18). Moreover, we found a population of cells that were notably longer than stationary cells and highly heterogeneous in length. We classified these cells under the predivision stage, as the elongation leads to cell division. Lastly, we classified cells with a clear division septum under the division stage. IMOD (3dmod Graph) (34) was used to measure cell dimensions in pixels. Pixel size was multiplied to the measured pixel counts to derive cell dimensions in micrometers.

### Subtomogram analysis.

Tomographic package I3 (0.9.9.3) was used for subtomogram analysis (35). A total of 1,164 T4SS machines from the whole cells (WT) and a total of 7,913 T4SS machines at cell poles (WT, *dotB*_E191K_, Δ*dotB*, and Δ*dotL* strains) were identified and extracted, respectively, as described previously (15). Four-by-4-by-4 binned subtomograms were used to classify particles into distinct conformations. The alignment proceeds iteratively with each iteration, consisting of three parts in which references and classification masks are generated, subtomograms are aligned and classified, and, finally, class averages are aligned to each other. The periplasmic complex displayed 13-fold symmetry; therefore, a 13-fold symmetry was imposed to assist the initial alignment process. Class average structures with similar features were selected together for an iterative cycle of alignment and classification until each conformation consists of highly homogeneous particles.

We used 2-by-2-by-2 binned subtomograms to obtain the cage-like collar structure and cytoplasmic complex structures (Fig. 4; see also Fig. S4D to K in the supplemental material).

### Resolution estimation.

The resolution estimation package ResMap was used to calculate local resolutions of the DotB-free and DotB-bound cytoplasmic complex average structures (36). The single-volume input method was used.

### 3D visualization and molecular modeling.

Outer membrane (OM), inner membrane (IM), and phosphate granules (P) were segmented using the EMAN 2.22 segmentation tool (37). Moreover, ribosomes from each bacterial cell were segmented from tomograms shown in Fig. 1A to C, averaged to ∼40-Å resolution, and mapped back to their original particle positions using EMAN 2.22. UCSF Chimera (38) and UCSF ChimeraX (39) were used to visualize the segmentations and subtomogram average structures in three dimensions (3D) and build an atomic model of the T4SS. The atomic model was built by docking the crystal structure of the L. pneumophila DotB complex (PDB entry 6GEB) (24). Video clips for the supplemental material were generated using UCSF ChimeraX and edited with iMovie.

### Direct tomogram analysis.

IMOD (34) was used to measure dimensions of L. pneumophila cells. The number of T4SS machines on each bacterium was carefully counted by examining the 4-by-4-by-4 binned SIRT reconstructed tomograms. Measurements were recorded in MS Excel for statistical analysis.

### Fluorescence microscopy imaging and processing.

Imaging of L. pneumophila expressing Dot-Icm fluorescent proteins was carried out by resuspension of 2-day heavy patches in water, after which they were spotted on a thin pad of 1% agarose, covered with a coverslip, and immediately imaged at room temperature. Fluorescence micrographs were captured using a Nikon Eclipse TE2000-S inverted microscope equipped with a Spectra X light engine from Lumencor, CoolSNAP EZ 20 MHz digital monochrome camera from Photometrics, and a Nikon Plan Apo 100× lens objective (1.4 numerical aperture) under the control of SlideBook 6.0 (Intelligent Imaging Innovations). Samples were imaged at 196 mW and 485 nm, with typical exposure times of 500 to 1,000 ms and 2-by-2 binning. Polarity scores were calculated with SlideBook by measuring the ratio between the variance and the mean of the fluorescence signal at regions of interest located between the pole and the cell center (15) and normalized to their wild-type controls.

## References

[1] Bhatty M, Laverde Gomez JA, Christie PJ 2013 The expanding bacterial type IV secretion lexicon. Res Microbiol 164:620–639. doi:10.1016/j.resmic.2013.03.012. 23542405 PMC3816095

[2] Christie PJ, Whitaker N, Gonzalez-Rivera C 2014 Mechanism and structure of the bacterial type IV secretion systems. Biochim Biophys Acta 1843:1578–1591. doi:10.1016/j.bbamcr.2013.12.019. 24389247 PMC4061277

[3] Goessweiner-Mohr N, Arends K, Keller W, Grohmann E 2013 Conjugative type IV secretion systems in Gram-positive bacteria. Plasmid 70:289–302. doi:10.1016/j.plasmid.2013.09.005. 24129002 PMC3913187

[4] Gonzalez-Rivera C, Bhatty M, Christie PJ 2016 Mechanism and function of type IV secretion during infection of the human host. Microbiol Spectr 4:10.1128/microbiolspec.VMBF-0024-2015. doi:10.1128/microbiolspec.VMBF-0024-2015. PMC4920089 27337453

[5] Christie PJ 2016 The mosaic type IV secretion systems. EcoSal Plus 7:10.1128/ecosalplus.ESP-0020-2015. doi:10.1128/ecosalplus.ESP-0020-2015. PMC5119655 27735785

[6] Chandran Darbari V, Waksman G 2015 Structural biology of bacterial type IV secretion systems. Annu Rev Biochem 84:603–629. doi:10.1146/annurev-biochem-062911-102821. 26034891

[7] Nagai H, Kubori T 2011 Type IVB secretion systems of *Legionella* and other Gram-negative bacteria. Front Microbiol 2:136. doi:10.3389/fmicb.2011.00136. 21743810 PMC3127085

[8] Vogel JP, Andrews HL, Wong SK, Isberg RR 1998 Conjugative transfer by the virulence system of *Legionella pneumophila*. Science 279:873–876. doi:10.1126/science.279.5352.873. 9452389

[9] Chung JM, Sheedlo MJ, Campbell AM, Sawhney N, Frick-Cheng AE, Lacy DB, Cover TL, Ohi MD 2019 Structure of the *Helicobacter pylori* Cag type IV secretion system. Elife 8:e47644. doi:10.7554/eLife.47644. 31210639 PMC6620104

[10] Sgro GG, Costa TRD, Cenens W, Souza DP, Cassago A, Coutinho de Oliveira L, Salinas RK, Portugal RV, Farah CS, Waksman G 2018 Cryo-EM structure of the bacteria-killing type IV secretion system core complex from *Xanthomonas citri*. Nat Microbiol 3:1429–1440. doi:10.108/s41564-018–0262-z. doi:10.1038/s41564-018-0262-z. 30349081 PMC6264810

[11] Low HH, Gubellini F, Rivera-Calzada A, Braun N, Connery S, Dujeancourt A, Lu F, Redzej A, Fronzes R, Orlova EV, Waksman G 2014 Structure of a type IV secretion system. Nature 508:550–553. doi:10.1038/nature13081. 24670658 PMC3998870

[12] Fronzes R, Schafer E, Wang L, Saibil HR, Orlova EV, Waksman G 2009 Structure of a type IV secretion system core complex. Science 323:266–268. doi:10.1126/science.1166101. 19131631 PMC6710095

[13] Chandran V, Fronzes R, Duquerroy S, Cronin N, Navaza J, Waksman G 2009 Structure of the outer membrane complex of a type IV secretion system. Nature 462:1011–1015. doi:10.1038/nature08588. 19946264 PMC2797999

[14] Redzej A, Ukleja M, Connery S, Trokter M, Felisberto-Rodrigues C, Cryar A, Thalassinos K, Hayward RD, Orlova EV, Waksman G 2017 Structure of a VirD4 coupling protein bound to a VirB type IV secretion machinery. EMBO J 36:3080–3095. doi:10.15252/embj.201796629. 28923826 PMC5916273

[15] Chetrit D, Hu B, Christie PJ, Roy CR, Liu J 2018 A unique cytoplasmic ATPase complex defines the *Legionella pneumophila* type IV secretion channel. Nat Microbiol 3:678–686. doi:10.1038/s41564-018-0165-z. 29784975 PMC5970066

[16] Ghosal D, Chang YW, Jeong KC, Vogel JP, Jensen GJ 2017 In situ structure of the *Legionella* Dot/Icm type IV secretion system by electron cryotomography. EMBO Rep 18:726–732. doi:10.15252/embr.201643598. 28336774 PMC5412798

[17] Ghosal D, Jeong KC, Chang YW, Gyore J, Teng L, Gardner A, Vogel JP, Jensen GJ 2019 Molecular architecture, polar targeting and biogenesis of the *Legionella* Dot/Icm T4SS. Nat Microbiol 4:1173–1182. doi:10.1038/s41564-019-0427-4. 31011165 PMC6588468

[18] Jeong KC, Ghosal D, Chang YW, Jensen GJ, Vogel JP 2017 Polar delivery of *Legionella* type IV secretion system substrates is essential for virulence. Proc Natl Acad Sci U S A 114:8077–8082. doi:10.1073/pnas.1621438114. 28696299 PMC5544279

[19] Hu B, Khara P, Song L, Lin AS, Frick-Cheng AE, Harvey ML, Cover TL, Christie PJ 2019 In situ molecular architecture of the *Helicobacter pylori* Cag type IV secretion system. mBio 10:e00849-19. doi:10.1128/mBio.00849-19. 31088930 PMC6520456

[20] Hu B, Khara P, Christie PJ 2019 Structural bases for F plasmid conjugation and F pilus biogenesis in *Escherichia coli*. Proc Natl Acad Sci U S A 116:14222–14227. doi:10.1073/pnas.1904428116. 31239340 PMC6628675

[21] Morado DR, Hu B, Liu J 2016 Using Tomoauto: a protocol for high-throughput automated cryo-electron tomography. J Vis Exp 107:e53608. doi:10.3791/53608. PMC4781705 26863591

[22] Khoshouei M, Pfeffer S, Baumeister W, Forster F, Danev R 2017 Subtomogram analysis using the Volta phase plate. J Struct Biol 197:94–101. doi:10.1016/j.jsb.2016.05.009. 27235783

[23] Vincent CD, Friedman JR, Jeong KC, Buford EC, Miller JL, Vogel JP 2006 Identification of the core transmembrane complex of the *Legionella* Dot/Icm type IV secretion system. Mol Microbiol 62:1278–1291. doi:10.1111/j.1365-2958.2006.05446.x. 17040490

[24] Prevost MS, Waksman G 2018 X-ray crystal structures of the type IVb secretion system DotB ATPases. Protein Sci 27:1464–1475. doi:10.1002/pro.3439. 29770512 PMC6153414

[25] Collins R, Karuppiah V, Siebert CA, Dajani R, Thistlethwaite A, Derrick JP 2018 Structural cycle of the *Thermus thermophilus* PilF ATPase: the powering of type IVa pilus assembly. Sci Rep 8:14022. doi:10.1038/s41598-018-32218-3. 30232337 PMC6145873

[26] McCallum M, Tammam S, Khan A, Burrows LL, Howell PL 2017 The molecular mechanism of the type IVa pilus motors. Nat Commun 8:15091. doi:10.1038/ncomms15091. 28474682 PMC5424180

[27] Kwak MJ, Kim JD, Kim H, Kim C, Bowman JW, Kim S, Joo K, Lee J, Jin KS, Kim YG, Lee NK, Jung JU, Oh BH 2017 Architecture of the type IV coupling protein complex of *Legionella pneumophila*. Nat Microbiol 2:17114. doi:10.1038/nmicrobiol.2017.114. 28714967 PMC6497169

[28] Vincent CD, Friedman JR, Jeong KC, Sutherland MC, Vogel JP 2012 Identification of the DotL coupling protein subcomplex of the *Legionella* Dot/Icm type IV secretion system. Mol Microbiol 85:378–391. doi:10.1111/j.1365-2958.2012.08118.x. 22694730 PMC3391322

[29] Merriam JJ, Mathur R, Maxfield-Boumil R, Isberg RR 1997 Analysis of the *Legionella pneumophila* fliI gene: intracellular growth of a defined mutant defective for flagellum biosynthesis. Infect Immun 65:2497–2501. doi:10.1128/IAI.65.6.2497-2501.1997. 9169800 PMC175352

[30] Buscher BA, Conover GM, Miller JL, Vogel SA, Meyers SN, Isberg RR, Vogel JP 2005 The DotL protein, a member of the TraG-coupling protein family, is essential for viability of *Legionella pneumophila* strain Lp02. J Bacteriol 187:2927–2938. doi:10.1128/JB.187.9.2927-2938.2005. 15838018 PMC1082803

[31] Hu B, Lara-Tejero M, Kong Q, Galán JE, Liu J 2017 In situ molecular architecture of the *Salmonella* type III secretion machine. Cell 168:1065–1074. doi:10.1016/j.cell.2017.02.022. 28283062 PMC5393631

[32] Mastronarde DN 2005 Automated electron microscope tomography using robust prediction of specimen movements. J Struct Biol 152:36–51. doi:10.1016/j.jsb.2005.07.007. 16182563

[33] Zheng SQ, Palovcak E, Armache JP, Verba KA, Cheng Y, Agard DA 2017 MotionCor2: anisotropic correction of beam-induced motion for improved cryo-electron microscopy. Nat Methods 14:331–332. doi:10.1038/nmeth.4193. 28250466 PMC5494038

[34] Kremer JR, Mastronarde DN, McIntosh JR 1996 Computer visualization of three-dimensional image data using IMOD. J Struct Biol 116:71–76. doi:10.1006/jsbi.1996.0013. 8742726

[35] Winkler H 2007 3D reconstruction and processing of volumetric data in cryo-electron tomography. J Struct Biol 157:126–137. doi:10.1016/j.jsb.2006.07.014. 16973379

[36] Kucukelbir A, Sigworth FJ, Tagare HD 2014 Quantifying the local resolution of cryo-EM density maps. Nat Methods 11:63–65. doi:10.1038/nmeth.2727. 24213166 PMC3903095

[37] Chen M, Dai W, Sun SY, Jonasch D, He CY, Schmid MF, Chiu W, Ludtke SJ 2017 Convolutional neural networks for automated annotation of cellular cryo-electron tomograms. Nat Methods 14:983–985. doi:10.1038/nmeth.4405. 28846087 PMC5623144

[38] Pettersen EF, Goddard TD, Huang CC, Couch GS, Greenblatt DM, Meng EC, Ferrin TE 2004 UCSF Chimera–a visualization system for exploratory research and analysis. J Comput Chem 25:1605–1612. doi:10.1002/jcc.20084. 15264254

[39] Goddard TD, Huang CC, Meng EC, Pettersen EF, Couch GS, Morris JH, Ferrin TE 2018 UCSF ChimeraX: meeting modern challenges in visualization and analysis. Protein Sci 27:14–25. doi:10.1002/pro.3235. 28710774 PMC5734306

