# Supplementary figures and images for: Analysis of Dot/Icm Type IVB Secretion System Subassemblies by Cryoelectron Tomography Reveals Conformational Changes Induced by DotB Binding
Source: mBio. 2020 Feb 18;11(1):e03328-19. doi: 10.1128/mBio.03328-19 (PMC7029142; doi:10.1128/mBio.03328-19)

A

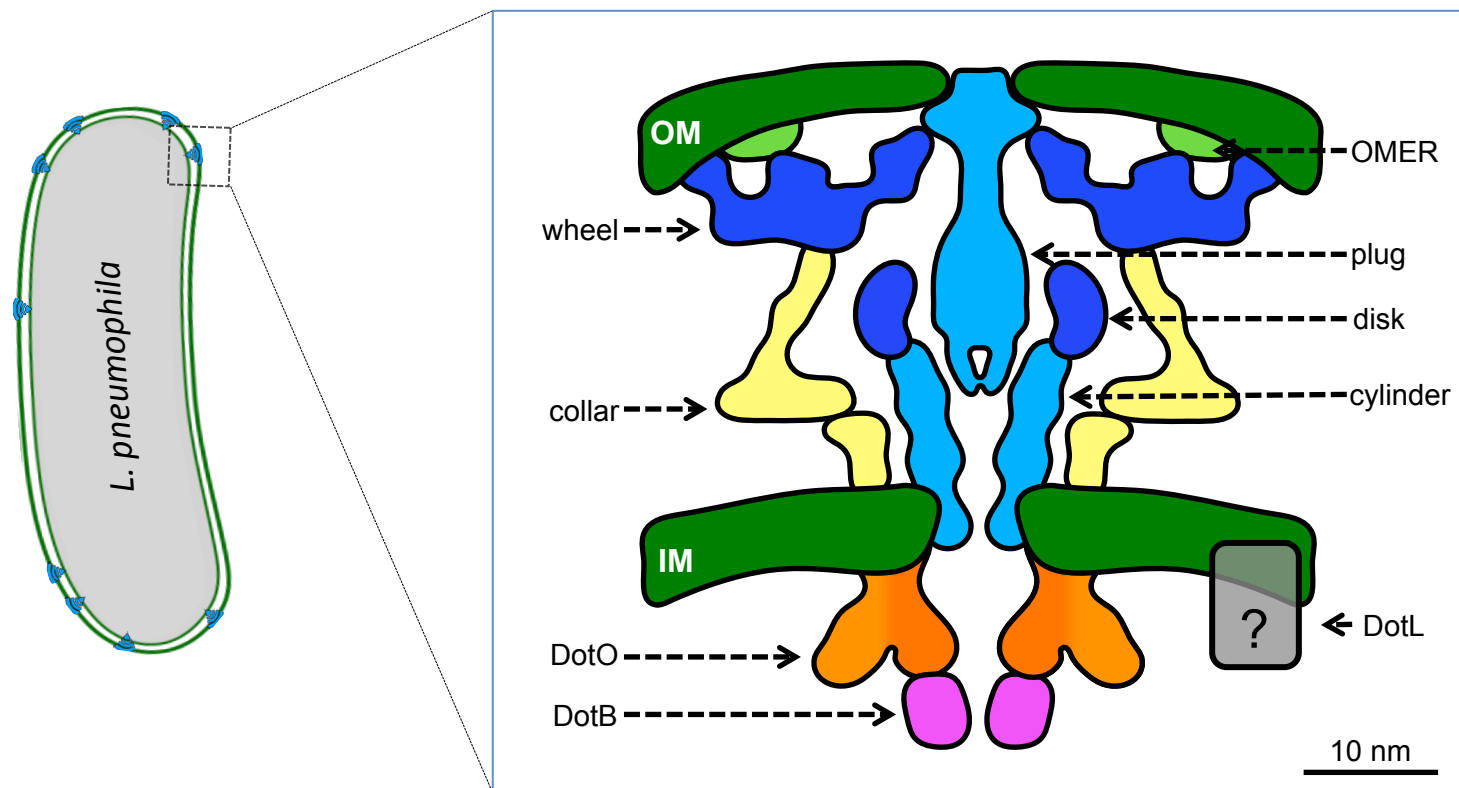

B

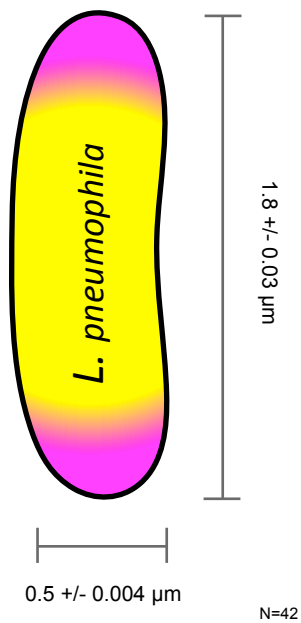

C

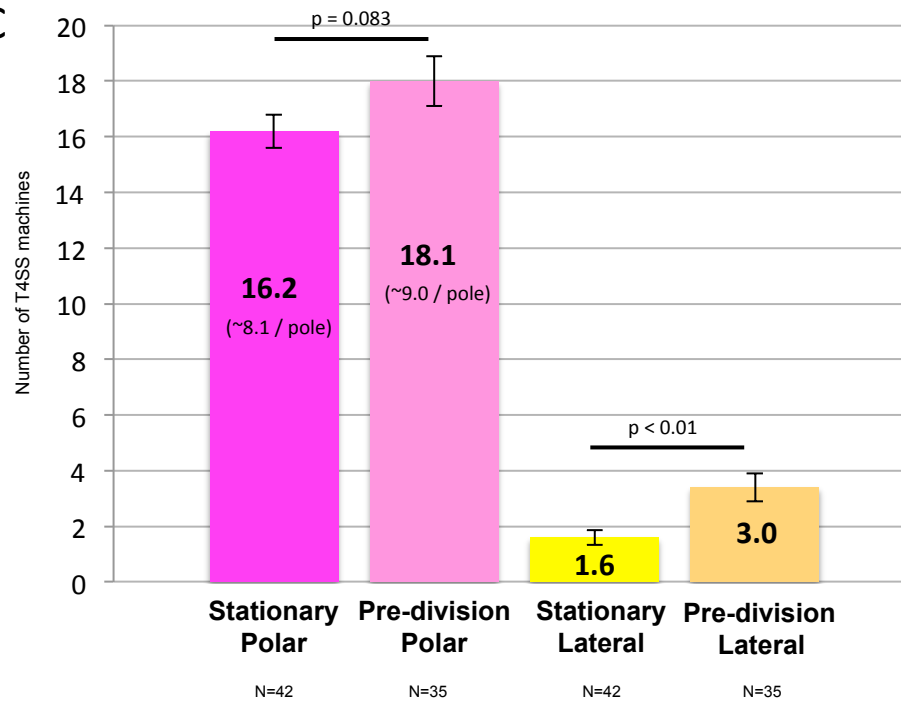

Supplement: FIG S1 [file mBio.03328-19-sf001.pdf]

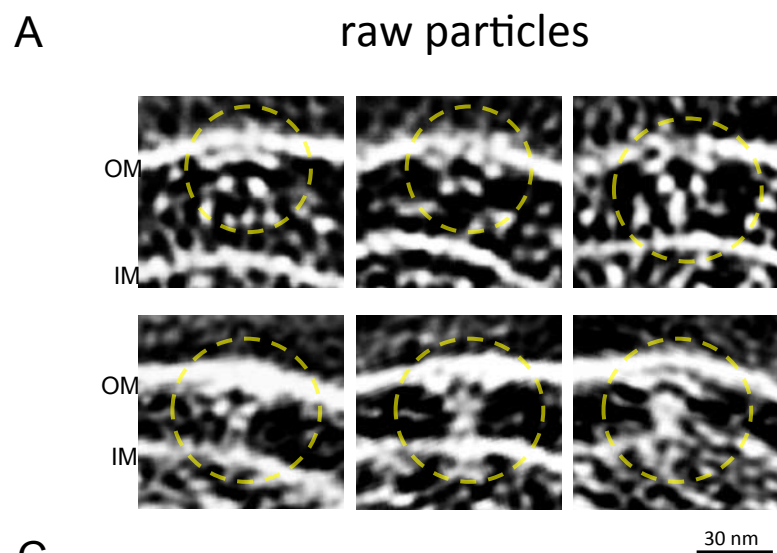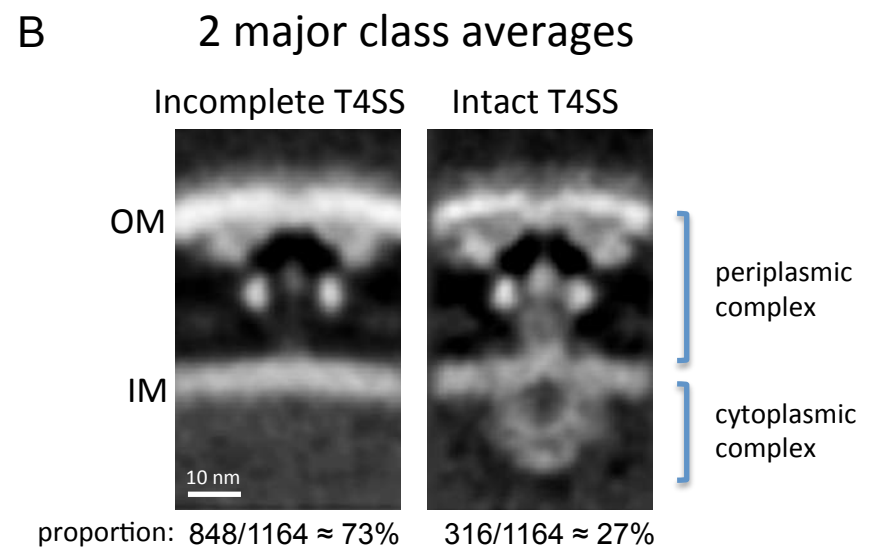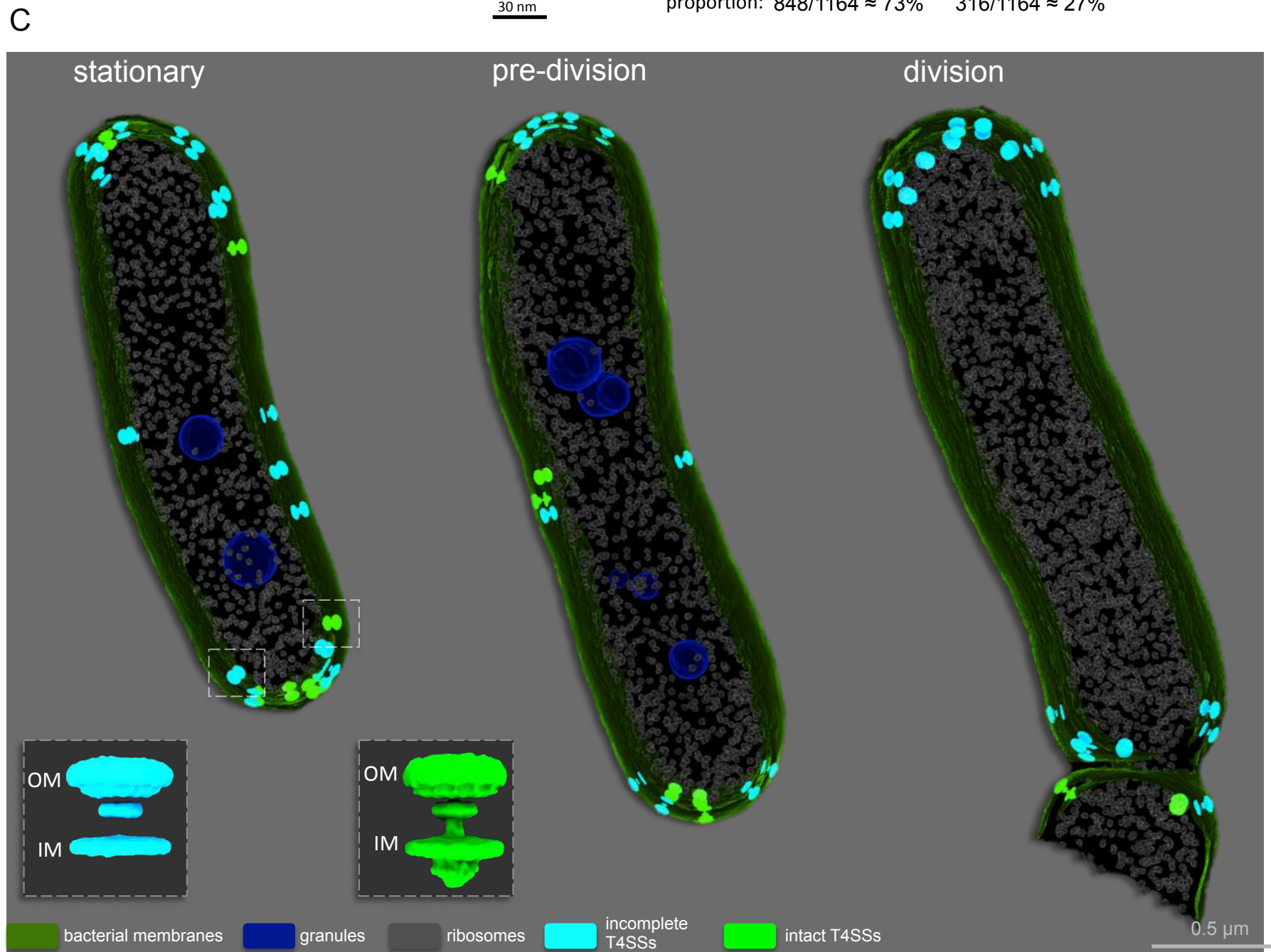

Supplement: FIG S2 [file mBio.03328-19-sf002.pdf]

Particle distributions

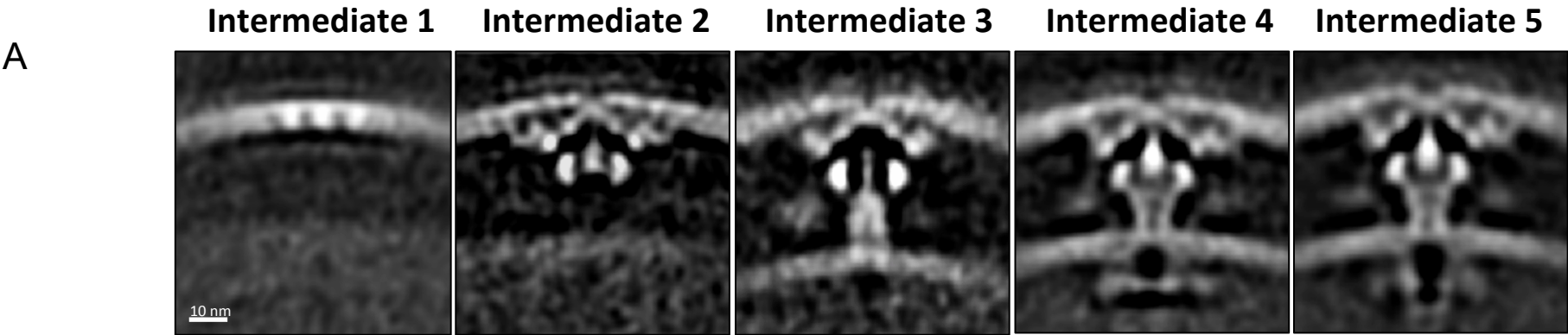

B

|                       |             |             |             |             |             |
|-----------------------|-------------|-------------|-------------|-------------|-------------|
| WT                    | 458 (26.8%) | 254 (14.9%) | 367 (21.5%) | 152 (8.9%)  | 475 (27.8%) |
| DotB <sub>E191K</sub> | 246 (20.5%) | 22 (1.8%)   | 123 (10.2%) | 315 (26.2%) | 495 (41.2%) |
| $\Delta dotB$         | 115 (22.2%) | 7 (1.3%)    | 99 (19.1%)  | 298 (57.4%) | 0 (0%)      |
| $\Delta dotL$         | 79 (19.7%)  | 8 (2%)      | 30 (7.5%)   | 120 (29.9%) | 164 (40.9%) |
| Total                 | 898         | 291         | 619         | 885         | 1134        |

Supplement: FIG S3 [file mBio.03328-19-sf003.pdf]

The Plug-Cylinder association

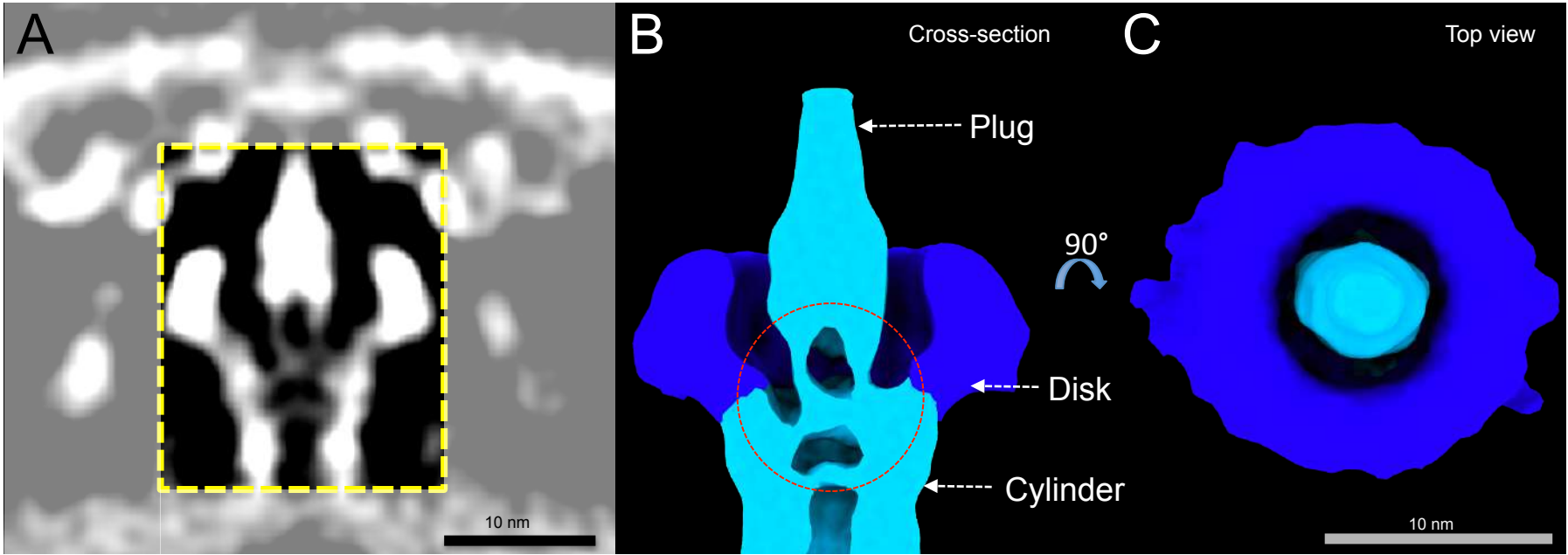

A 13-fold symmetric case-like structure of the collar

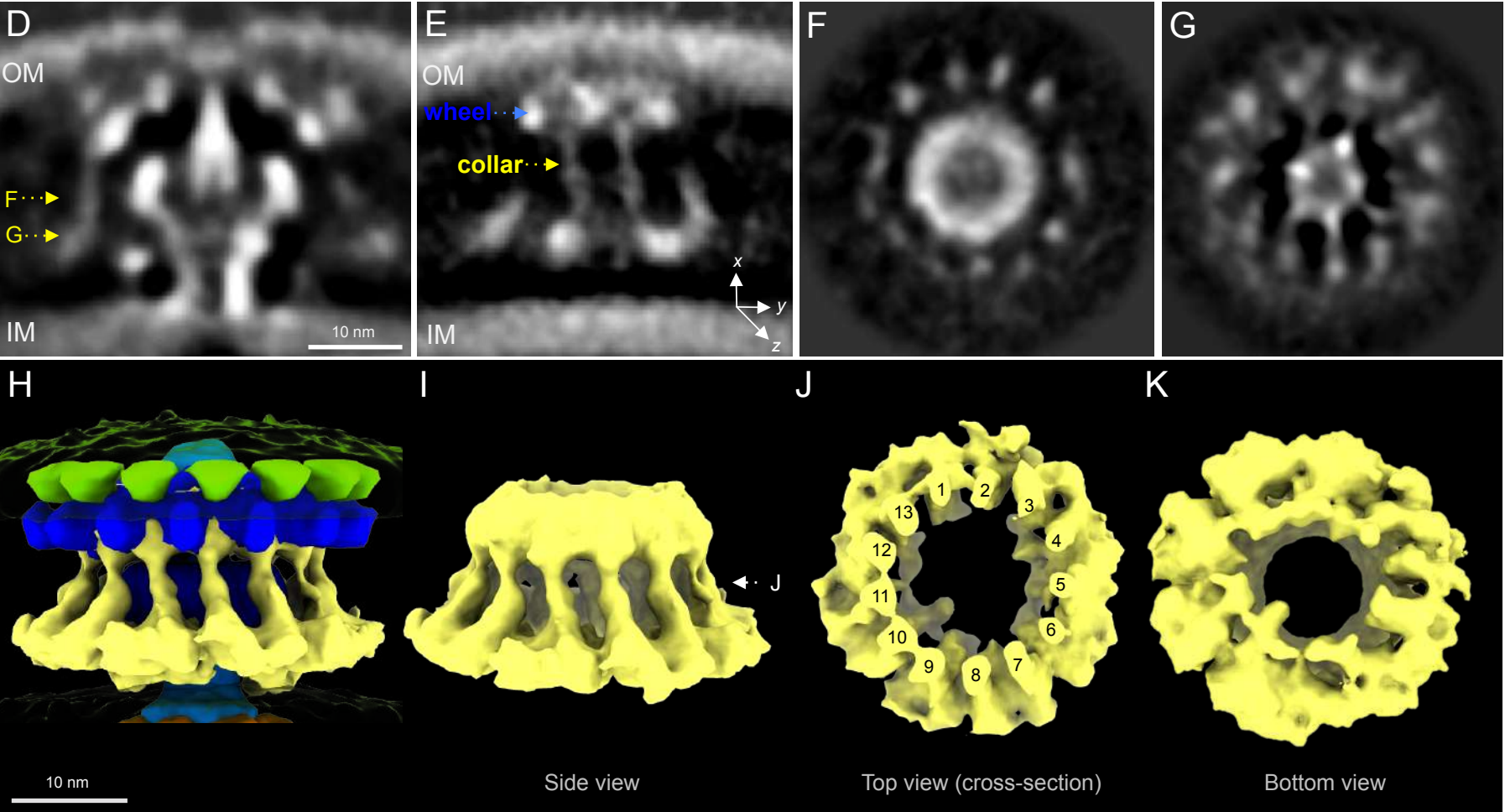

Supplement: FIG S4 [file mBio.03328-19-sf004.pdf]

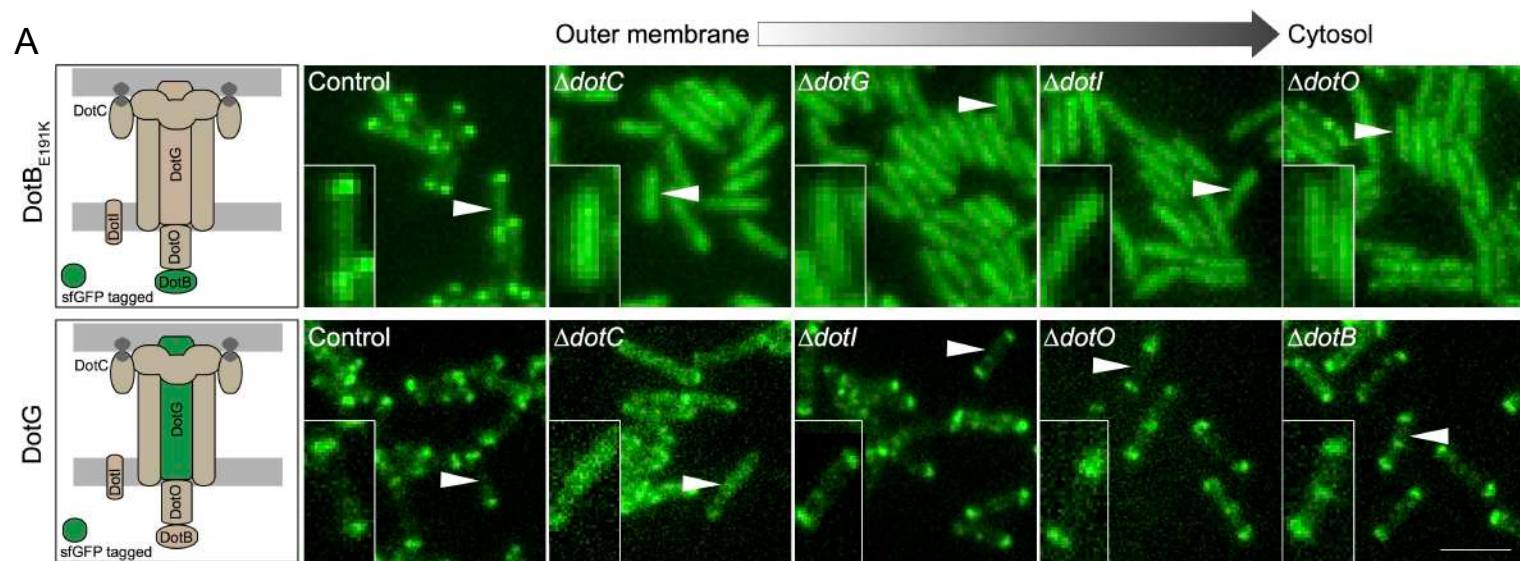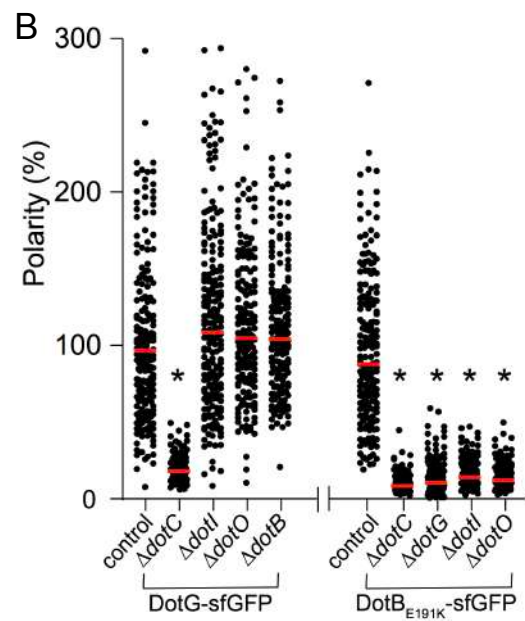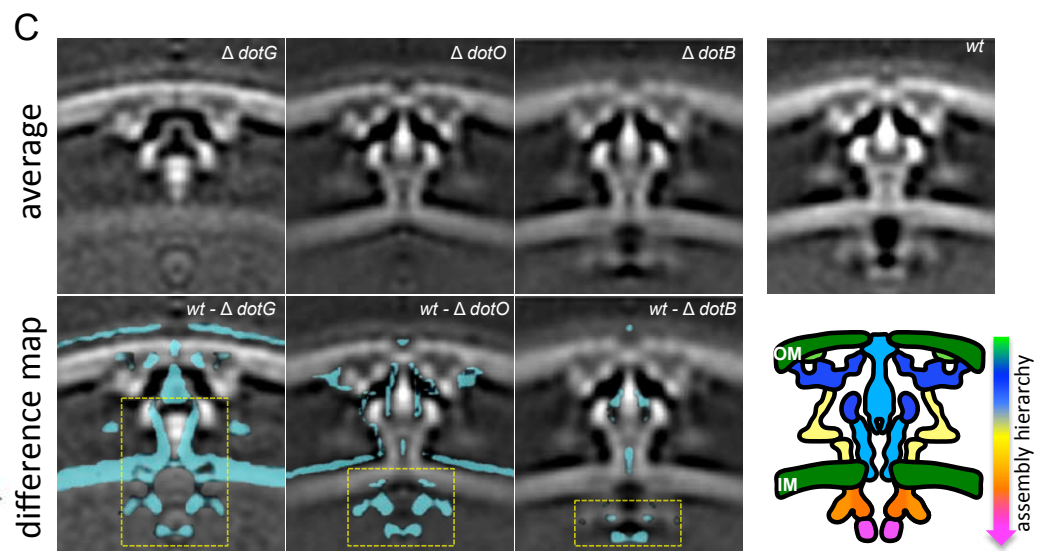

Supplement: FIG S5 [file mBio.03328-19-sf005.pdf]

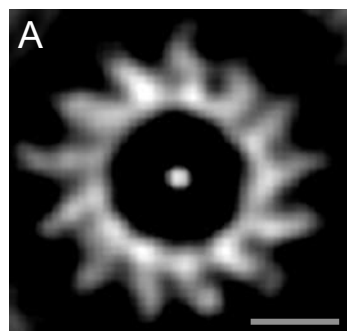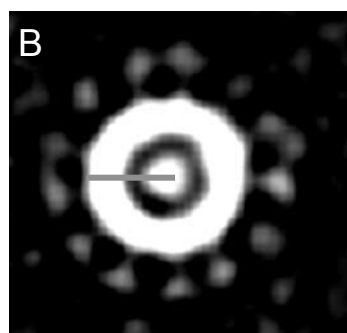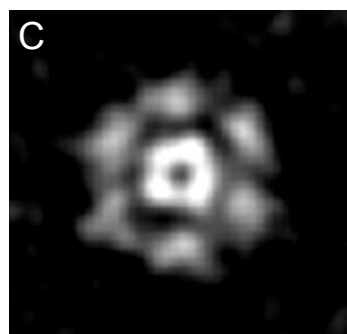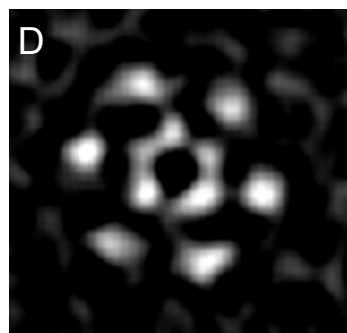

Bin4, global alignment

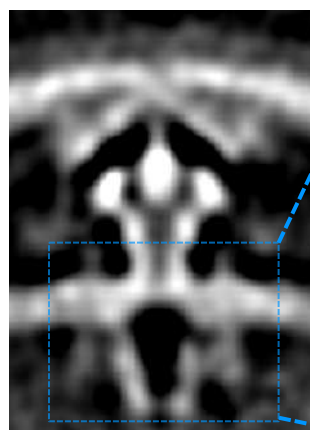

A →  
B →  
C →  
D →

Bin2, focused refinement

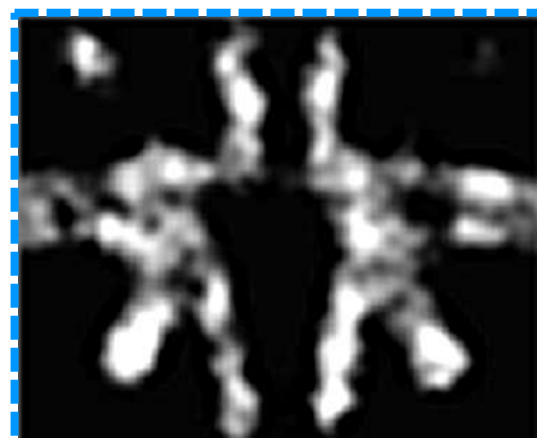

E →  
F →  
G →  
H →  
I →

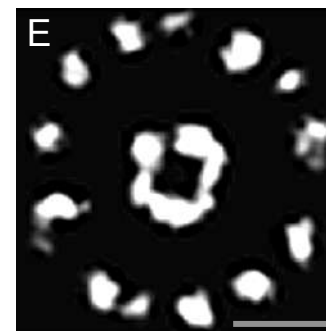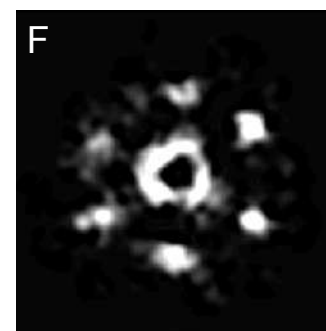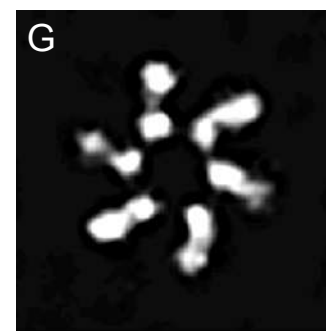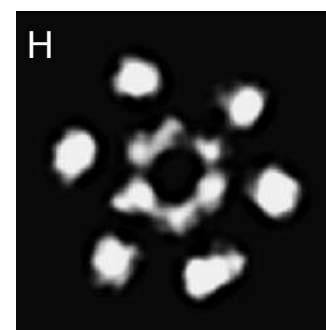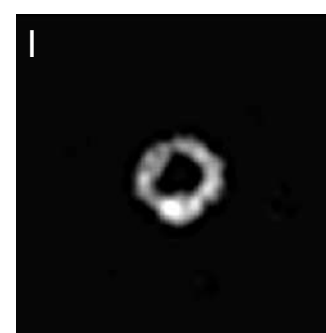

Supplement: FIG S6 [file mBio.03328-19-sf006.pdf]

Inactive

Active

A

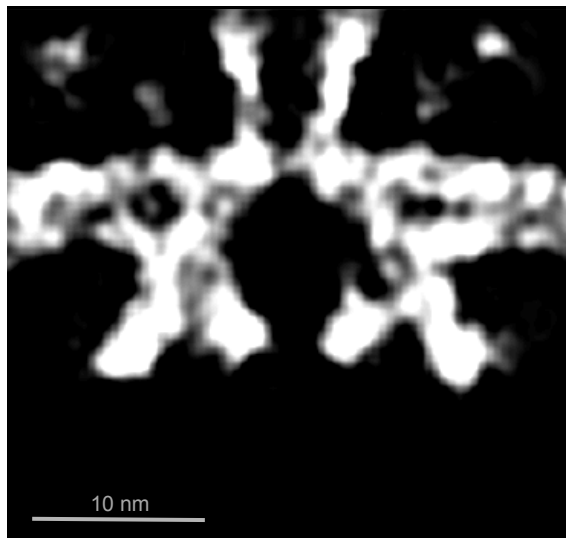

B

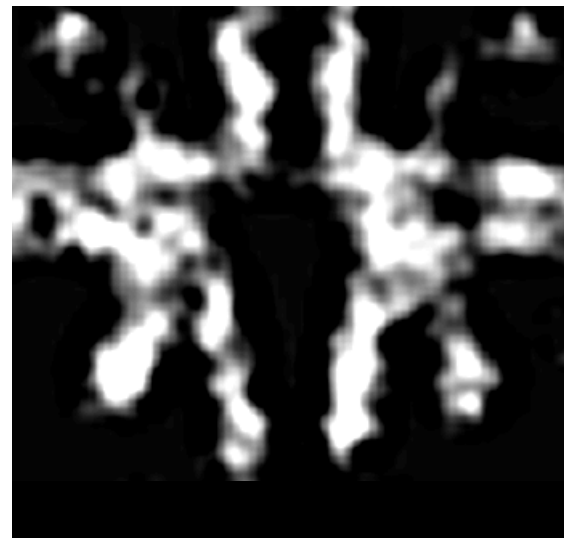

C

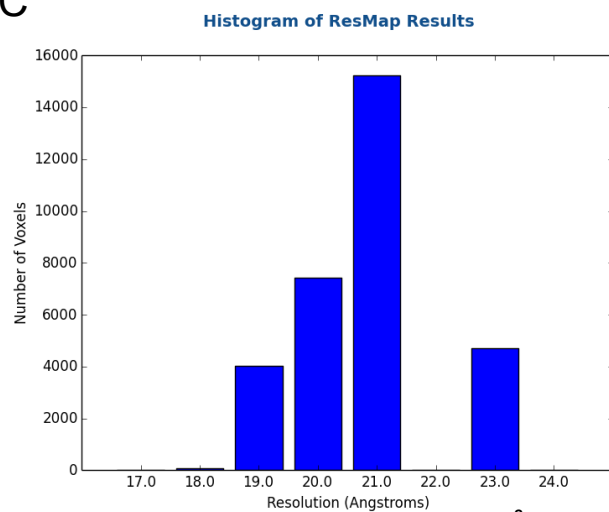

Mean resolution = 20.80 Å

D

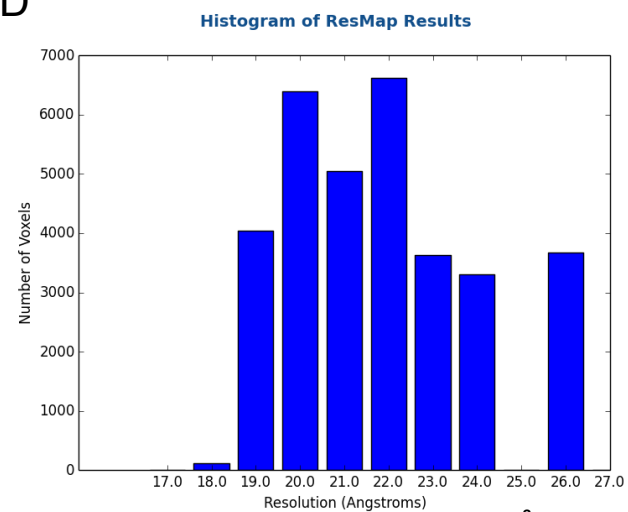

Mean resolution = 21.83 Å

E

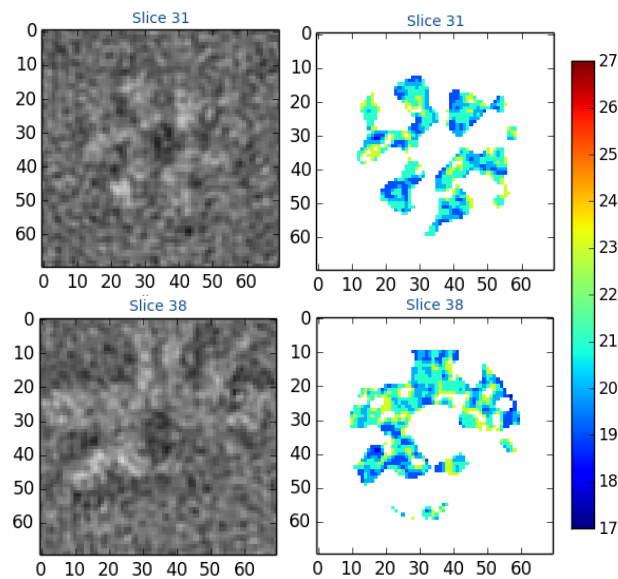

F

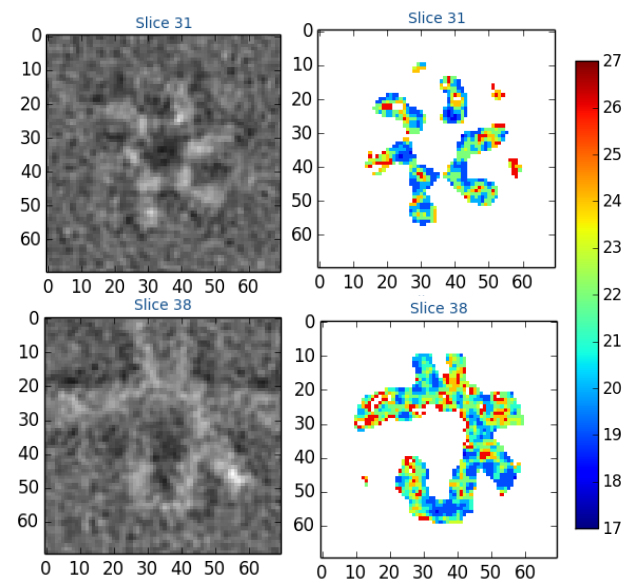

Supplement: FIG S7 [file mBio.03328-19-sf007.pdf]
