# Supplementary material for: Analysis of Dot/Icm Type IVB Secretion System Subassemblies by Cryoelectron Tomography Reveals Conformational Changes Induced by DotB Binding
Source: mBio. 2020 Feb 18;11(1):e03328-19. doi: 10.1128/mBio.03328-19 (PMC7029142; doi:10.1128/mBio.03328-19)
Supplement: TABLE S1 [file mBio.03328-19-st001.pdf]

Supplementary Information Table 1. Strains used in this study

Genotype	Parent strain	Description/comments	Source/reference
LPO1 (wild type)		StrR, hsdR	(*1*)
LPO2 ΔdotB		StrR, hsdR, thyA	(*2*)
LPO1 $dotB_{E191K}$*-sfGFP*		StrR, hsdR	(*3*)
LPO1 dotG-sfGFP		StrR, hsdR	(*3*)
LPO1 dotG-sfGFP ΔdotC	CR2710	StrR, hsdR	This study
LPO1 dotG-sfGFP ΔdotI	CR419	StrR, hsdR	This study
LPO1 dotG-sfGFP ΔdotO	CR2710	StrR, hsdR	This study
LPO1 dotG-sfGFP ΔdotB	CR2710	StrR, hsdR	This study
LPO1 $dotB_{E191K}$*-sfGFP ΔdotC*	CR2701	StrR, hsdR	(*3*)
LPO1 $dotB_{E191K}$*-sfGFP ΔdotG*	CR2701	StrR, hsdR	(*3*)
LPO1 $dotB_{E191K}$*-sfGFP ΔdotI*	CR2701	StrR, hsdR	(*3*)
LPO1 $dotB_{E191K}$*-sfGFP ΔdotO*	CR2701	StrR, hsdR	(*3*)
LPO1 $dotB_{E191K}$*-sfGFP ΔdotL*	CR2701	StrR, hsdR	(*3*)

1. K. H. Berger, R. R. Isberg, Two distinct defects in intracellular growth complemented by a single genetic locus in Legionella pneumophila. *Mol Microbiol* **7**, 7-19 (1993).
2. M. Matthews, C. R. Roy, Identification and subcellular localization of the Legionella pneumophila IcmX protein: a factor essential for establishment of a replicative organelle in eukaryotic host cells. *Infect Immun* **68**, 3971-3982 (2000).
3. D. Chetrit, B. Hu, P. J. Christie, C. R. Roy, J. Liu, A unique cytoplasmic ATPase complex defines the Legionella pneumophila type IV secretion channel. *Nat Microbiol* **3**, 678-686 (2018).
